# Association between Yo-yo intermittent recovery test, physiological markers of endurance performance, and buffering capacity in professional soccer players

**DOI:** 10.1590/1414-431X2026e15230

**Published:** 2026-10-12

**Authors:** R.C.A. Ferreira, V.O. Silvino, L.V. Vilhena, M.A.P. dos Santos, D.M.L. do Prado

**Affiliations:** 1 Find Your Pace Lab, São Paulo, SP, Brasil; 2 Departamento de Biofísica e Fisiologia, Núcleo de Estudos em Fisiologia Aplicada ao Desempenho e è Saúde (NEFADS), Universidade Federal do Piauí, Teresina, PI, Brasil; 3 Rede Nordeste de Biotecnologia (RENORBIO), Universidade Federal do Piauí, Teresina, PI, Brasil; 4 Programa de Pós-graduação Multicêntrico em Ciências Fisiológicas (PPGMCF), Sociedade Brasileira de Fisiologia (SBFis), Universidade Federal do Piauí, Teresina, PI, Brasil; 5 Laboratório de Avaliação e Condicionamento em Reumatologia (LACRE), Hospital das Clínicas, Faculdade de Medicina, Universidade de São Paulo, São Paulo, SP, Brasil

**Keywords:** Soccer, Intermittent endurance, V̇O_2max_, Maximal aerobic speed, Ventilatory threshold, Buffering capacity

## Abstract

The Yo-Yo Intermittent Recovery Test Level 1 (YYIR1) is widely recognized as a reliable tool for assessing intermittent endurance and aerobic capacity in team sports athletes. This study aims to verify the association between YYIR1 and physiological markers of endurance performance in professional soccer players, as well as the relationship between metabolic buffering capacity, metabolic acidosis tolerance, and YYIR1 total distance. Twenty-six professional soccer athletes (26.3±4.98 years, 76.4±8.96 kg, 178±6.52 cm, 54.1±4.85 mL·kg^−1^·min^−1^ V̇O_2max_) performed the YYIR1 and underwent a cardiopulmonary exercise test (CPET) on a treadmill in order to assess their ventilatory thresholds (aerobic threshold (AT), respiratory compensation point (RCP), and maximal oxygen consumption (V̇O_2max_)), buffering capacity, and tolerance to metabolic acidosis. Correlation analyses revealed that the YYIR1 distance was significantly associated with maximal aerobic speed (r=0.542, P=0.006) and V̇O_2max_ (r=0.492, P=0.015), but not with AT or RCP. Position-specific differences were observed in relative acidosis tolerance capacity, with midfielders presenting higher values than defenders (P=0.018, η^2^=0.294), whereas no significant differences were noted for relative buffering or YYIR1 distance across positions. In conclusion, maximal aerobic speed and V̇O_2max_ were the strongest predictors of YYIR1 performance in professional soccer players, outperforming submaximal thresholds such as AT and RCP. Moreover, positional differences in acidosis tolerance were identified, as midfielders demonstrated greater relative acidosis tolerance capacity compared to defenders.

## Introduction

Aerobic fitness is a critical determinant of soccer performance, enabling players to sustain repeated high-intensity efforts, maintain overall match intensity, and recover rapidly between bouts of maximal activity (1). Given the intermittent nature of soccer, with frequent accelerations, decelerations, and changes in direction, the capacity to perform and recover from repeated intense efforts is a distinguishing characteristic of elite players (2).

The Yo-Yo Intermittent Recovery Test Level 1 (YYIR1) has become one of the most popular and practical field tests for assessing a player's ability to repeatedly perform intense intermittent exercise, effectively reflecting the physical demands experienced during match play (3). In addition to providing insights into players' aerobic and anaerobic recovery capacity, YYIR1 performance has been shown to correlate with total distance covered and high-intensity running distance during matches, further highlighting its relevance in performance monitoring (4).

However, it is important to note that aerobic fitness is a multifaceted construct involving not only field performance but also various physiological markers of endurance capacity. These include the aerobic threshold (AT), respiratory compensation point (RCP), and maximal oxygen consumption (V̇O_2max_), each of which contributes uniquely to an athlete's endurance performance (5). In this context, cardiopulmonary exercise testing (CPET) has been widely used as a comprehensive tool to assess these physiological markers, providing objective and individualized information essential for training prescription and performance optimization (6).

A deeper physiological perspective also considers the body's ability to manage metabolic acidosis induced by high-intensity exercise. For instance, the relative buffering capacity (RBC), also known as the isocapnic buffering period, defined as the interval between AT and RCP, reflects the body's capacity to buffer hydrogen ions and delay fatigue onset. In addition, relative acidosis tolerance capacity (RAC), or the hypocapnic hyperventilation period, defined as the phase from RCP to maximal effort, represents the athlete's tolerance to progressive metabolic acidosis and the ability to sustain maximal hyperventilation (7). These buffering and tolerance capacities are crucial for maintaining performance during repeated high-intensity actions typical of soccer matches.

Despite the practical value of YYIR1 in soccer, there is a lack of studies exploring the combined relationships between laboratory-based physiological markers, metabolic buffering capacity, and field-based intermittent endurance performance. Additionally, potential differences across playing positions, each with distinct physical and physiological demands, have not been fully elucidated.

Therefore, the primary aim of this study was to investigate the association between physiological markers of endurance capacity (AT, RCP, and V̇O_2max_), as well as the relationship between RBC and RAC, and YYIR1 performance in professional soccer players, considering positional differences. As a secondary objective, we evaluated the relationships between physiological markers of endurance performance and total distance covered in the YYIR1. We hypothesized that players with higher laboratory-based endurance markers and superior buffering and tolerance capacities achieve better YYIR1 performance, irrespective of their playing position.

## Material and Methods

### Subjects

Twenty-six professional soccer athletes (26.3±4.98 years, 76.4±8.96 kg, 178±6.52 cm, 54.1±4.85 mL·kg^−1^·min^−1^) participated in the study. All athletes played in a second division league and had professional work contracts with their club. The athletes participated in regular systematic training three to five times a week in addition to competitive matches. Players were briefed on the study details and test protocols without receiving explicit information regarding the study's objectives. This study was conducted according to the principles of the Declaration of Helsinki and was approved in 2024 by the institutional review board of the Universidade Federal do Piauí, Brazil, under protocol 6.737.475. All subjects signed the informed consent form according to resolution 466/12 of the National Health Council.

### Experimental design

This was a cross-sectional study. All evaluations were carried out during the first 2 preseason weeks. Tests were conducted in the morning, with coaching staff instructed that the players should avoid vigorous training sessions the day before. Additionally, players were advised: 1) to abstain from high-intensity physical activities 24 h prior to testing, 2) to refrain from taking analgesic or anti-inflammatory medication 48 h before testing, and 3) to avoid consuming stimulants like caffeine on the test days. Firstly, the athletes performed the YYIR1 and, after seven days, they performed a cardiopulmonary exercise test (CPET). Players who did not complete the tests at each time point for the YYIR1 or CPET were excluded from the study.

#### Yo-yo intermittent recovery test level 1 (YYIR1)

The YYIR1 consists of shuttle runs over a distance of 40 m (2×20 m with a 180-degree change of direction), performed with progressively increasing speed, with intervals of 10 s of active recovery (8). Active recovery consists of walking for a distance of 10 m (2×5 m) at the end of each 40-m run. The course (20 m of testing plus five meters of recovery) was marked by cones. The initial speed of the test was 10 km/h, which was increased to 13 km/h in the first four stages (total distance of 160 m). For the next seven stages (160-440 m), the speed was increased from 13 to 14 km/h. Afterwards, there were increments of 0.5 km/h in speed every eight runs. The total distance covered in the test was used as a performance index. The test was terminated when the athlete failed to reach the cone in the initial portion of the 20 m twice consecutively or due to voluntary exhaustion.

#### Cardiopulmonary exercise testing (CEPT)

The CPET was carried out using a programmable treadmill manufactured by Inbramed (model ATL, Brazil). Gas exchange and ventilatory variables were continuously measured breath-by-breath with a metabolic analyzer system (Cortex, MetaLyzer IIIB R2, Germany). The CPET was carried out using an incremental protocol to determine peak oxygen consumption (V̇O_2max_). Prior to the test, participants performed a 5-min warm-up at 7 km/h. The initial speed was set at 8 km/h, with increments of 1 km/h every 60 s at a 1% incline. The testing environment was indoors, maintaining standard conditions with temperatures ranging between 22-24°C and approximately 45-60% relative humidity. The following criteria were used to define maximal effort: 1) the occurrence of a plateau in oxygen consumption, 2) peak heart rate (HR) ≥95% age-predicted maximum or subjective exhaustion, or 3) maximal respiratory exchange ratio (RER)≥1.10 (9).

#### Aerobic threshold

The AT was defined as the break point between the increase in the carbon dioxide output and VO_2_ (V-slope) or the point at which VE/ VO_2_ reached its minimum value and began to rise without a concomitant rise in VE/VCO_2_ (minute ventilation/carbon dioxide production) (10).

#### Respiratory compensation point

The RCP was defined as the point at which VE/VCO_2_ reached its minimum value and began to rise, and the highest value of PetCO_2_ before its progressive fall (11).

#### Peak oxygen consumption

The peak oxygen consumption was defined as the maximum VO_2_ attained during the exercise period in which the subject reached exhaustion (analog scale from Borg's scale of perceived exertion).

#### Buffering capacity

In this study, the RBC, also called isocapnic buffering period, was evaluated as the ratio of buffering capacity within performance up to the RCP. Specifically, RBC represents the difference between the running speed at AT and RCP, expressed as a percentage, as seen in the following equation (12), where S_RCP_ and S_AT_ stand for the running speed at respiratory compensation point and anaerobic threshold, respectively.

$$RBC=\frac{\left(S_{RCB}-S_{AT}\right)}{S_{RCB}}\times 100$$ (Eq. 1)

#### Relative acidosis tolerance capacity

The RAC, also called hypocapnic hyperventilation, expressed as a percentage, was calculated in accordance with the following equation (13), where S_RCP_ and S_peak_ stand for the running speed at respiratory compensation point and at peak of exercise, respectively.

$$RAC=\frac{\left(S_{peak}-S_{RCP}\right)}{S_{peak}}\times 100$$ (Eq. 2)

### Statistical analysis

Normal distribution and homogeneity of variance of the data were tested using the Shapiro-Wilk and Levene's tests, respectively. The data were analyzed using one-way ANOVA to compare means across playing positions (defenders, forwards, midfielders). Effect sizes were calculated using eta-squared (η^2^), with interpretations guided by Cohen's benchmarks (η^2^: 0.01=small, 0.06=medium, 0.14=large) (14). Pearson's correlation coefficient was used to examine the relationships between physiological markers of endurance performance (e.g., V̇O_2max_, maximal aerobic speed (MAS), and ventilatory thresholds) and total distance covered in the YYIR1. The magnitude of the correlation was interpreted as follows: trivial (<0.1), small (≥0.1 and <0.3), moderate (≥0.3 and <0.5), large (≥0.5 and <0.7), very large (≥0.7 and <0.9), and nearly perfect (≥0.9). The significance level adopted for the statistical procedures was P≤0.05. Statistical analysis was performed using the Jamovi software for Windows (15).

## Results

The characteristics of the participants are presented in Table 1. No significant differences were observed between playing positions.

**Table 1:** Description of study participants.

Variable	Defenders (n=10)	Midfielders (n=10)	Forwards (n=6)	Overall (n=26)
Age (years)	26.2±4.5	27.0±6.4	25.7±4.5	26.3±5.0
Stature (cm)	180.3±6.7	175.7±4.9	176.2±8.0	178.0±6.5
Body weight (kg)	80.5±8.3	73.3±5.2	74.5±13.1	76.4±9.0

Data are reported as means and SD.

Table 2 shows the parameters obtained after the CPET and the YYIR1. Defenders reached the respiratory compensation point at significantly higher speed than midfielders during the CPET. In contrast, defenders presented higher respiratory exchange ratio than midfield players at peak of exercise during the CPET. No significant differences were observed in YYIR1 distance between defenders, midfielders, and forwards (782.00±171.39, 791.11±176.38, and 736.00±197.18 m, respectively; P=0.850, η^2^=0.015), indicating a small effect size.

**Table 2:** Cardiopulmonary exercise testing (CPET) results of the participants according to playing position.

	Defenders (n=10)	Midfielders (n=10)	Forwards (n=6)	Overall (n=26)	P	η^2^
Ventilatory anaerobic threshold						
Time (min)	8.00±1.83	7.15±1.40	8.42±1.62	7.77±1.64	0.291	0.102
Speed (km/h)	9.90±1.91	9.00±1.70	9.83±1.47	9.54±1.73	0.470	0.063
VO_2_ (mL·kg^−1^·min^−1^)	32.94±6.81	33.69±5.23	37.7±6.19	34.3±6.15	0.317	0.095
VO_2_ (L/min)	2.60±0.57	2.48±0.42	2.8±0.56	2.60±0.51	0.535	0.053
HR (bpm)	139.60±18.40	138.89±17.49	152.00±11.11	142±17.0	0.342	0.097
Respiratory compensation point						
Time (min)	13.10±1.43^a^	11.50±1.31	12.67±1.47	12.4±1.53	0.049	0.231
Speed (km/h)	15.00±1.33^a^	13.20±1.48	13.83±1.47	14.0±1.59	0.030	0.263
VO_2_ (mL·kg^−1^·min^−1^)	46.97±5.62	45.02±4.03	47.70±5.51	46.4±4.96	0.536	0.053
VO2 (L/min)	3.76±0.63	3.27±0.38	3.53±0.58	3.52±0.56	0.144	0.155
HR (bpm)	164.88±11.68	163.20±12.18	175.80±7.01	167±11.8	0.130	0.184
Peak of exercise						
Time (min)	16.20±0.89	15.47±1.79	15.83±1.63	15.8±1.44	0.546	0.051
Speed (km/h)	17.90±0.99	17.40±1.17	17.00±1.26	17.5±1.14	0.304	0.098
VO_2_ (mL·kg^−1^·min^−1^)	54.01±4.87	54.25±4.95	53.97±5.57	54.1±4.85	0.992	0.001
VO_2_ (L/min)	4.34±0.68^b^	3.97±0.46	3.66±1.15	4.04±1.76	0.210	0.127
HR (bpm)	179.22±7.80	183.50±4.88	187.80±9.55	183±7.62	0.119	0.183
RER (units)	1.11±0.09^b^	1.27±0.14	1.24±0.10	1.20±0.13	0.012	0.321

Data are reported as means±SD. ^a^P<0.05 compared to midfielders; ^b^trend towards higher value compared to midfielders; ANOVA.

No differences were observed in relative buffering capacity when comparing defenders, midfielders, and forwards (34.37±9.28, 31.78±10.15, and 29.00±6.34%, respectively), as seen in Figure 1A (P=0.524, η^2^=0.055), representing a small-to-medium effect size. However, Figure 1B shows that midfield players had significantly greater relative acidosis tolerance capacity compared to defenders (24.71±6.76 *vs* 16.22±5.48, respectively), while forwards did not present significantly different values (18.71±4.45) compared to the other groups of players (P=0.018, η^2^=0.294), indicating a large effect size.

**Figure 1:**
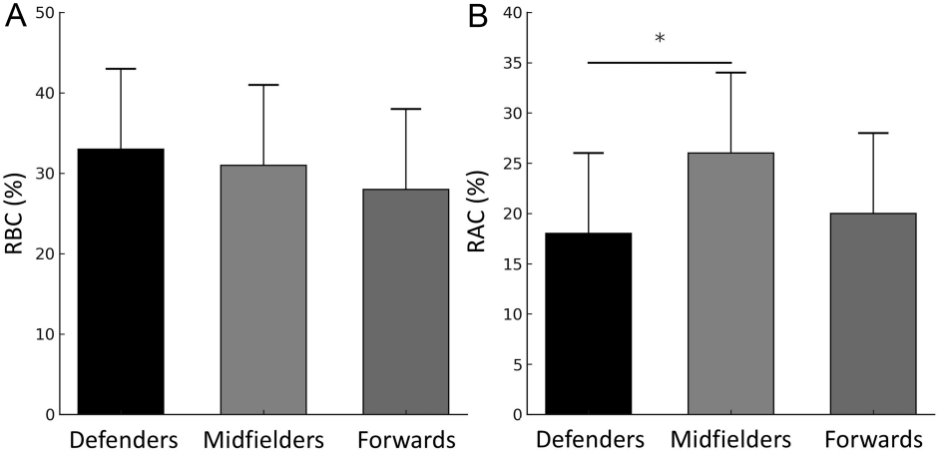
Comparisons between defenders, midfielders, and forwards regarding relative buffering capacity (**A**) and relative acidosis tolerance capacity (**B**). Data are reported as means and SD. *P<0.05; ANOVA. RBC: relative buffering capacity; RAC: relative acidosis tolerance capacity.

Finally, no significant correlation was found between YYIR1 distance and speed and VO_2_ at AT (Figure 2A and B, respectively), as well as speed and VO_2_ at RCP (Figure 2C and D, respectively). However, there was a statistically significant moderate-to-large correlation between total distance covered in the YYIR1 and MAS (Figure 2E) and V̇O_2max_ (Figure 2F).

**Figure 2:**
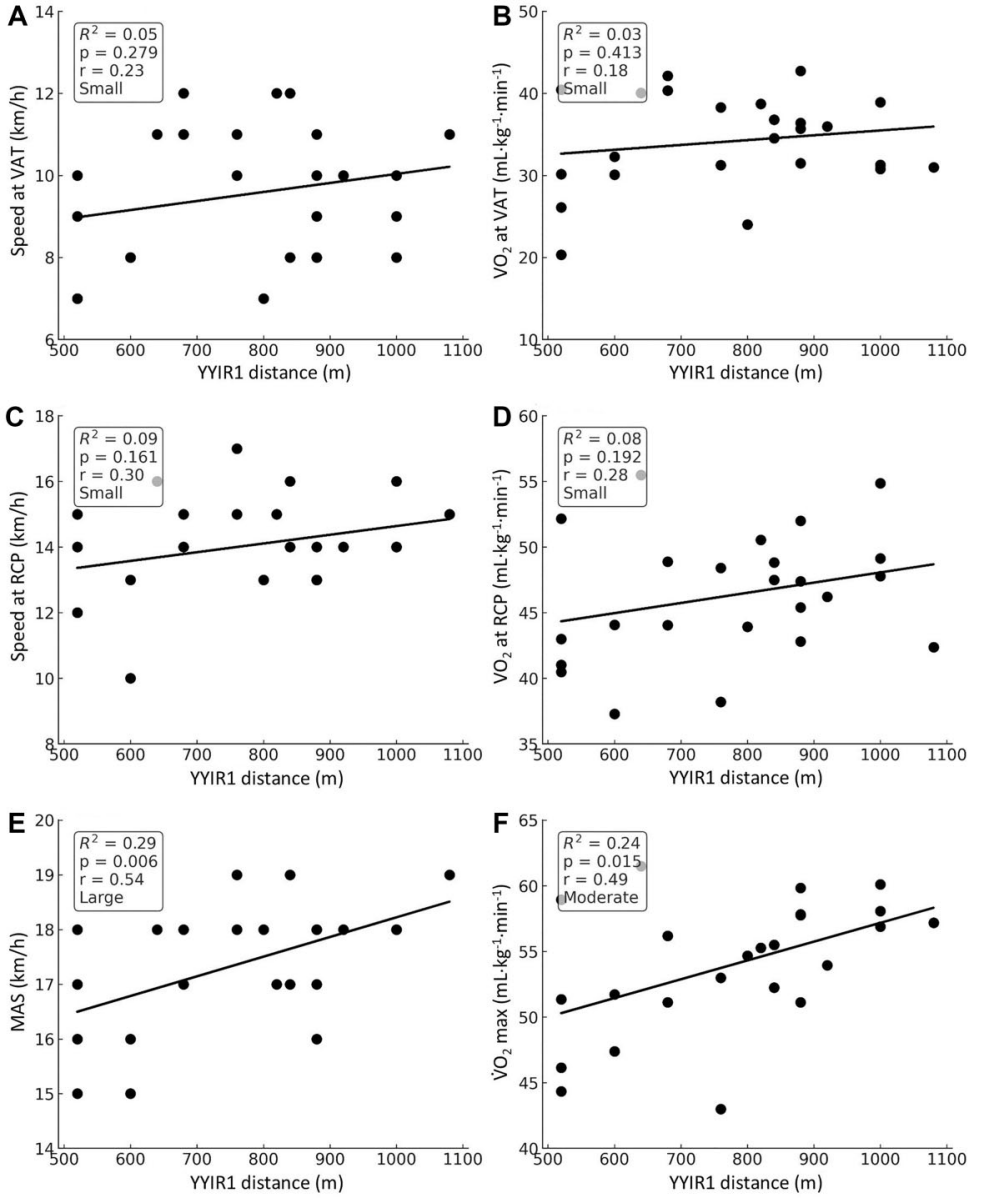
Pearson's correlation between YYIR1 total distance with speed and oxygen consumption (VO_2_) at ventilatory anaerobic threshold (**A** and **B**, respectively), speed and VO_2_ at respiratory compensation point (**C** and **D**, respectively), and speed and V̇O_2max_ at peak exercise (**E** and **F**, respectively). AT: aerobic threshold; MAS: maximal aerobic speed; RCP: respiratory compensation point; V̇O_2max_: maximal oxygen consumption; YYIR1: Yoyo intermittent recovery test level 1.

## Discussion

This study investigated the association between performance in the YYIR1 and key physiological markers of endurance performance, namely ventilatory thresholds, MAS, and V̇O_2max_, as well as buffering and acidosis tolerance capacity in professional soccer players. Our findings revealed that MAS and V̇O_2max_ were the strongest predictors of YYIR1 performance, while no significant correlations were observed with submaximal markers such as AT and RCP. Additionally, we observed position-specific differences in acidosis tolerance, with midfielders displaying higher relative acidosis tolerance capacity than defenders.

The positive correlations between YYIR1 distance, V̇O_2max_, and MAS reinforced the notion that maximal aerobic capacity remains a crucial determinant of intermittent endurance performance in soccer. These results are consistent with previous research highlighting the importance of V̇O_2max_ in supporting high-intensity intermittent efforts and total distance covered during competitive matches (8,16). Given that YYIR1 simulates the intermittent demands of match play, its strong association with MAS and V̇O_2max_ confirmed its validity as a functional field-based assessment of aerobic capacity in elite soccer players.

Interestingly, the absence of significant correlations between YYIR1 performance and submaximal thresholds (AT and RCP) suggests that YYIR1 may not be sensitive to moderate-intensity endurance markers. This contrasts with some findings in endurance sports, where submaximal thresholds are often predictive of performance (17-19). However, in the context of soccer, the intermittent and high-intensity nature of match play likely places a premium on an athlete's ability to sustain near-maximal efforts rather than operate efficiently at submaximal intensities. This may explain the stronger relationship with MAS and V̇O_2max_.

Our findings also point to position-related physiological adaptations, particularly with regard to acid-base balance during exercise. While no differences were detected in relative buffering capacity among defenders, midfielders, and forwards, midfielders demonstrated significantly higher relative acidosis tolerance capacity compared to defenders. The greater RAC values found in midfielders are likely explained by position-specific physiological adaptations derived from their higher exposure to high-intensity running during training and competitive play. This aligns with match analyses indicating that midfielders typically engage in a greater volume of high-intensity running and transitional actions, requiring enhanced tolerance to exercise-induced metabolic acidosis. This supports the notion that positional roles in soccer influence metabolic adaptations and training priorities (20).

Previous studies have found mixed results regarding V̇O_2max_ differences across playing positions in soccer. Some report higher values in midfielders and defenders compared to forwards (21,22), while others find minimal positional variation (23,24). Our data show no significant V̇O_2max_ differences by position, suggesting that while positional demands may shape certain metabolic characteristics, such as RAC, overall aerobic capacity remains relatively homogeneous among outfield players in professional squads, likely due to uniformity in training regimens. The lack of significant differences in YYIR1 distance across playing positions further supports this interpretation. Although the YYIR1 test reflects aerobic endurance and recovery capacity, its discriminatory power may be limited in homogeneous high-level groups where physical preparation is standardized across roles. Nonetheless, the test remains valuable for monitoring individual fitness changes over time or assessing recovery from injury.

This study had some limitations that should be acknowledged. First, the relatively small sample size and the absence of goalkeepers limit the generalizability of the findings across all playing positions. Second, the cross-sectional design precludes causal inferences regarding the relationship between endurance markers and YYIR1 performance. Future studies should consider longitudinal designs to evaluate how training interventions targeting MAS, V̇O_2max_, and buffering capacity affect YYIR1 outcomes and match performance. Moreover, investigations involving female athletes, players from different competitive levels, and the integration of biochemical or genetic markers (e.g., ACTN3, ACE polymorphisms) could provide a more comprehensive understanding of the physiological adaptations underlying endurance performance in soccer.

## Conclusions

This study demonstrated that maximal aerobic capacity, represented by V̇O_2max_ and MAS, is strongly associated with performance in the YYIR1 in professional soccer players. In contrast, submaximal physiological markers such as AT and RCP showed no significant relationship with YYIR1 outcomes, suggesting that the test primarily reflects the ability to sustain high-intensity intermittent efforts rather than moderate-intensity endurance. Additionally, differences in relative acidosis tolerance capacity were identified according to playing position, with midfielders exhibiting significantly higher values compared to defenders. In contrast, no significant positional differences were found in relative buffering capacity or in the distance covered in the YYIR1 test.

## Data Availability

The datasets generated and/or analyzed during the current study are available upon request from the corresponding author.
